# Factors Producing Variation in Postoperative Surveillance After Distal Radius Open Reduction and Internal Fixation: A Multi-surgeon Cohort Study

**DOI:** 10.7759/cureus.107887

**Published:** 2026-04-28

**Authors:** Alexis Watson, Shreya Sharma, Kyle DeRoma, Christina Grannie, Asiya Falak, Mark Shima

**Affiliations:** 1 Carle Illinois College of Medicine, University of Illinois Urbana-Champaign, Urbana, USA; 2 Orthopaedic Surgery, Carle Foundation Hospital, Urbana, USA

**Keywords:** distal radius fractures, healthcare disparities, interdisciplinary teams, open reduction and internal fixation, postoperative care

## Abstract

Introduction

Postoperative follow-up after open reduction and internal fixation (ORIF) of distal radius fracture varies widely, yet determinants of follow-up utilization are poorly understood. The purpose of this study was to evaluate the independent influence of surgeon-level factors versus patient sociodemographics on follow-up utilization. We hypothesized that, in a modern integrated health system, surgeon-specific practice infrastructure would be the primary driver of variability in follow-up.

Methods

This retrospective cohort study (2019-2024) analyzed adult distal radius ORIF patients within a single health system. Multivariable negative binomial regression evaluated patient demographics, comorbidities, insurance, and operating surgeon as predictors of postoperative follow-up frequency. Secondary outcomes included complications, including nerve, tendon, wound-related issues, and hardware removal. Surgeon identities were de-identified, with low-volume providers grouped as "Other".

Results

Among 731 patients (mean age: 55.3 years), the mean surgeon follow-up was 1.95±1.79 visits (range: 0-11). While 190 (26%) patients had zero encounters with the operating surgeon, all 731 (100%) patients received clinical surveillance via advanced practice providers (APPs). Multivariable regression identified female sex (incidence rate ratio (IRR): 1.26; 95% CI: 1.08-1.46; z=2.997; p=0.003) and private insurance (IRR: 1.31; 95% CI: 1.06-1.63; z=2.494; p=0.013) as independent predictors of higher utilization. Surgeon identity was the strongest predictor of follow-up frequency, with significant variation across providers (p<0.05). Although complications occurred in only 53 patients (7.3%), they were strongly associated with increased visits (IRR: 2.47; 95% CI: 2.14-2.85; z=12.37; p<0.001); however, complication rates did not fully account for the observed provider-level variability. Age, race, comorbidities, and smoking status were not significant predictors (p>0.05).

Conclusion

Postoperative follow-up utilization after distal radius ORIF demonstrates marked variability and is influenced by both patient- and provider-level factors. Surgeon-specific practice patterns play a substantial role in determining follow-up frequency.

## Introduction

Distal radius fractures are one of the most common types of fractures encountered in orthopedic hand surgery practices, accounting for approximately 20% of pediatric fractures and up to 18% of fractures in the elderly population [1]. Open reduction and internal fixation (ORIF) is frequently performed to treat unstable distal radius fractures [2,3]. Although ORIF of the distal radius is a common and routine procedure in which implants are retained, complications such as infection, hardware irritation, and the need for secondary surgery may occur [4-6]. The risk of complications makes postoperative monitoring essential for patients. The Centers for Disease Control and Prevention (CDC) recommends postoperative surveillance for superficial surgical site infections within 30 days of surgery and monitoring for deep infections within 90 days following procedures involving implants [7]. However, postoperative follow-up schedules after distal radius ORIF remain largely dependent on surgeon discretion and preference, with no standardized guidelines despite the potential for future complications [2,3,8].

In addition to variability in scheduling practices, patient adherence to postoperative follow-up represents an important determinant of surgical outcomes. Multiple studies have examined factors associated with postoperative follow-up attendance in orthopedic office visits. For example, a higher Area Deprivation Index (ADI) has been weakly correlated with reduced clinic attendance after treatment for a distal radius fracture [9]. Other retrospective reviews have identified associations between missed appointments and demographic or socioeconomic factors, including sex, race, insurance status, income, education level, and relationship status [10-13]. One study reported that there is a reported association between no-show rates in orthopedic injuries and the male sex, patients aged 26-35 years, self-reported races other than White, employment reported as disabled, household income less than $25,000, education below completion of high school, uninsured, Medicaid insured, and patients reporting their marital status as single [11]. Similarly, tobacco use, greater travel distance, lack of private insurance, and higher comorbidity burden have been linked to decreased likelihood of follow-up [10,13].

Despite these findings, there remains an inconsistency across studies regarding which patient-level determinants most strongly predict postoperative follow-up behavior. Furthermore, current literature often overlooks the role of clinical practice infrastructure, specifically the integration of advanced practice providers (APPs) such as physician assistants and nurse practitioners, in shaping follow-up volume [14]. In many modern orthopedic practices, APPs manage a significant portion of postoperative surveillance, and their presence may fundamentally alter the frequency and nature of patient visits independently of the surgeon's primary clinical intent [15,16]. Limited research has evaluated the full extent to which these surgeon-level and infrastructure-related factors may independently influence follow-up utilization after ORIF of the distal radius [17-19]. Understanding these predictors is critical for identifying patients at risk of inadequate postoperative surveillance and improving care delivery.

The purpose of this study was to evaluate whether patient demographics, insurance type, and operating surgeon independently predict postoperative follow-up utilization, specifically defined as face-to-face visits with the attending surgeon. We hypothesized that surgeon-level practice infrastructure, including the utilization of APPs and established clinical habits, would significantly influence variability in these surgeon-directed visits. Understanding these drivers is a critical first step toward standardizing postoperative guidelines to reduce patient burden and healthcare costs.

## Materials and methods

Study design and population

An institutional review board (IRB)-approved retrospective cohort study was conducted of adult patients (≥18 years) who underwent distal radius ORIF within the Carle Health System in Urbana, Illinois, between January 2019 and January 2024. All medical records were de-identified prior to analysis to ensure patient confidentiality. Patients were identified using Current Procedural Terminology (CPT) codes for ORIF of the distal radius, namely, CPT 25607 for extra-articular distal radius fractures, CPT 25608 for two-part intra-articular distal radius fractures, and CPT 25609 for comminuted intra-articular distal radius fractures with three or more fragments, along with procedural coding and operative records. Exclusion criteria included patients who underwent revision procedures at outside institutions or had incomplete electronic medical record (EMR) documentation. This study was approved by the Carle Foundation Hospital Institutional Review Board (approval number: 25CRU4225) with a waiver of informed consent.

Variables and outcomes

Patient-level variables extracted from the EMR included age, sex, race, diabetes status, smoking history (never, former, current), insurance type (private, Medicare, Medicaid, uninsured), and operating surgeon. Surgeon identities were deidentified, and surgeons performing fewer than 30 distal radius ORIF procedures during the study period were grouped into a single "Other" category to preserve statistical stability. The participating surgeons operated within various clinical models, ranging from solo-practitioner setups with direct scheduling to team-based models involving dedicated APPs. While these infrastructure differences (e.g., presence of APP support) likely influenced total clinic throughput, the primary focus was placed on surgeon-directed visits to isolate the impact of an individual provider's decision-making and practice infrastructure on care delivery. While APP encounters provided essential clinical surveillance for all patients, they were not included in follow-up visit counts to avoid masking the variation in surgeon-specific practice habits.

The primary outcome was the number of postoperative follow-up visits, defined as the number of postoperative clinic visits with the primary operating surgeon who performed the ORIF of the distal radius, as recorded in the EMR within two years of the index procedure. Follow-up visit utilization was analyzed as a count variable. Secondary outcomes included postoperative complications that arose only after the index procedure, defined as documented carpal tunnel syndrome, complex regional pain syndrome, tendon irritation or rupture, surgical site infection, or reoperation for hardware removal. 

Statistical analysis

Descriptive statistics summarized patient demographics, clinical characteristics, and postoperative follow-up visit utilization. Continuous variables were reported as means with standard deviations or medians with interquartile ranges, as appropriate, and categorical variables were summarized using frequencies and percentages. Continuous variables included age at the time of surgery and the number of follow-up visits. Categorical variables included gender, race, diabetes status, smoking status, insurance type, and operating surgeon.

Because postoperative follow-up visit counts showed overdispersion, multivariable negative binomial regression was used to evaluate factors associated with the number of follow-up visits. Results were reported as incidence rate ratios (IRRs) with corresponding 95% confidence intervals (CI). Covariates included age, gender, race, diabetes status, smoking status, insurance type, and surgeon category (de-identified as Surgeons 1 through 6 and Other Surgeons). The surgeon category was utilized as a proxy for both individual clinical preference and the associated practice infrastructure (such as dedicated staffing and workflow patterns) inherent to that specific provider's clinic.

Statistical significance was defined as a two-sided p-value of <0.05. Model fit was assessed by examining dispersion and regression diagnostics, with the Pearson dispersion statistic close to 1 (X^2^/df=1.13), indicating minimal residual overdispersion and appropriate model fit.

To assess whether postoperative complications influenced follow-up utilization, a secondary negative binomial regression model was run with complication status as the sole predictor of the number of follow-up visits.

## Results

The study cohort included 731 patients with a mean age of 55.3±16.8 years (range: 18-89). The cohort consisted of 508 (69.5%) male patients and 223 (30.5%) female patients, with the majority of patients, 623 (85.2%), self-identifying as White. Diabetes was present in 65 patients (8.9%). Smoking status included 409 (56%) never smokers, 201 (27.5%) former smokers, and 121 (16.6%) current smokers. Insurance coverage varied, with 336 (46%) privately insured, 255 (34.9%) covered by Medicare, 86 (11.8%) covered by Medicaid, and 54 (7.4%) uninsured (Table 1). 

**Table 1:** Population characteristics, surgeon distribution, and surgeon-specific postoperative follow-up utilization (n=731)

Variable	Patient count (n) or mean(±SD)	Percentage (%)
Total number of patients in cohort	731	100%
Gender		
Male patients	508	69.5%
Female patients	223	30.5%
Race		
White patients	623	85.2%
Black/African American patients	32	4.4%
None of the above/other patients	45	6.2%
Hispanic patients	1	0.1%
Asian patients	23	3.1%
American Indian/Alaskan Native patients	1	0.1%
Patient refused/unknown patients	6	0.8%
Average patient age (years)	55.30±16.78	-
Diabetes status		
Diabetes	65	8.9%
No diabetes	666	91.1%
Smoking status		
Never smoker	409	56%
Former smoker	201	27.5%
Current smoker	121	16.6%
Insurance type		
Private	336	46%
Medicare	255	34.9%
Medicaid	86	11.8%
Uninsured	54	7.4%
Clinical outcomes		
Avg. surgeon follow-ups	1.95±1.79	-
Patients with complications	53	7.3%
Number of operations by surgeon		
Surgeon 1	91	12.4%
Surgeon 2	37	5.1%
Surgeon 3	71	9.7%
Surgeon 4	167	22.8%
Surgeon 5	98	13.4%
Surgeon 6	202	27.6%
Other Surgeons	65	8.9%

**Figure 1:**
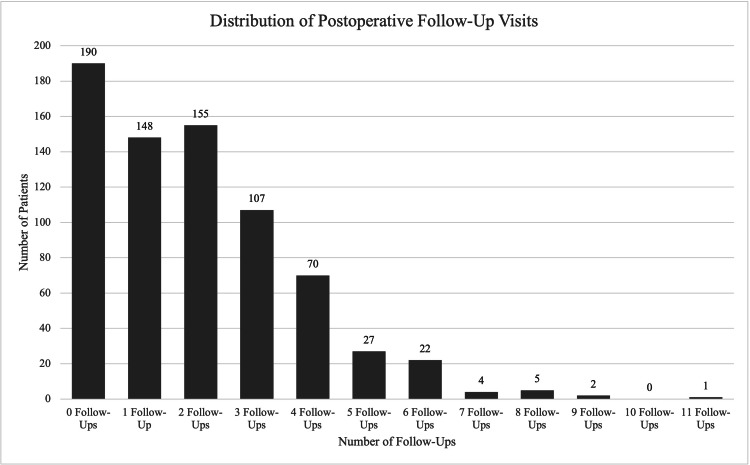
Distribution of postoperative follow-up visit counts after distal radius open reduction and internal fixation

Patients had a mean of 1.95±1.79 postoperative follow-up visits with their operating surgeon, with a median of 2 (IQR: 0-3) and a range of 0-11. A total of 190 patients (26%) had no recorded postoperative follow-up visits with their operating surgeon documented, which was the most frequently reported number of follow-up visits (Figure 1). It is important to note that while these patients lacked an attending-level encounter, 100% of the cohort had at least one documented encounter with an APP in the surgeon's office, ensuring baseline postoperative surveillance was maintained. Surgeon-specific follow-up visit frequency varied substantially across surgeons, with marked differences in postoperative utilization patterns. The distribution of postoperative visits with the operating surgeon is as follows: 190 patients (26%) had 0 follow-ups, 148 patients (20.2%) had one follow-up, 155 patients (21.2%) had two follow-ups, 107 patients (14.6%) had three follow-ups, 70 patients (9.6%) had four follow-ups, 27 patients (3.7%) had five follow-ups, 22 patients (3%) had six follow-ups, and the remaining 12 patients (1.64%) had greater than six follow-ups (Figure 1).

**Figure 2:**
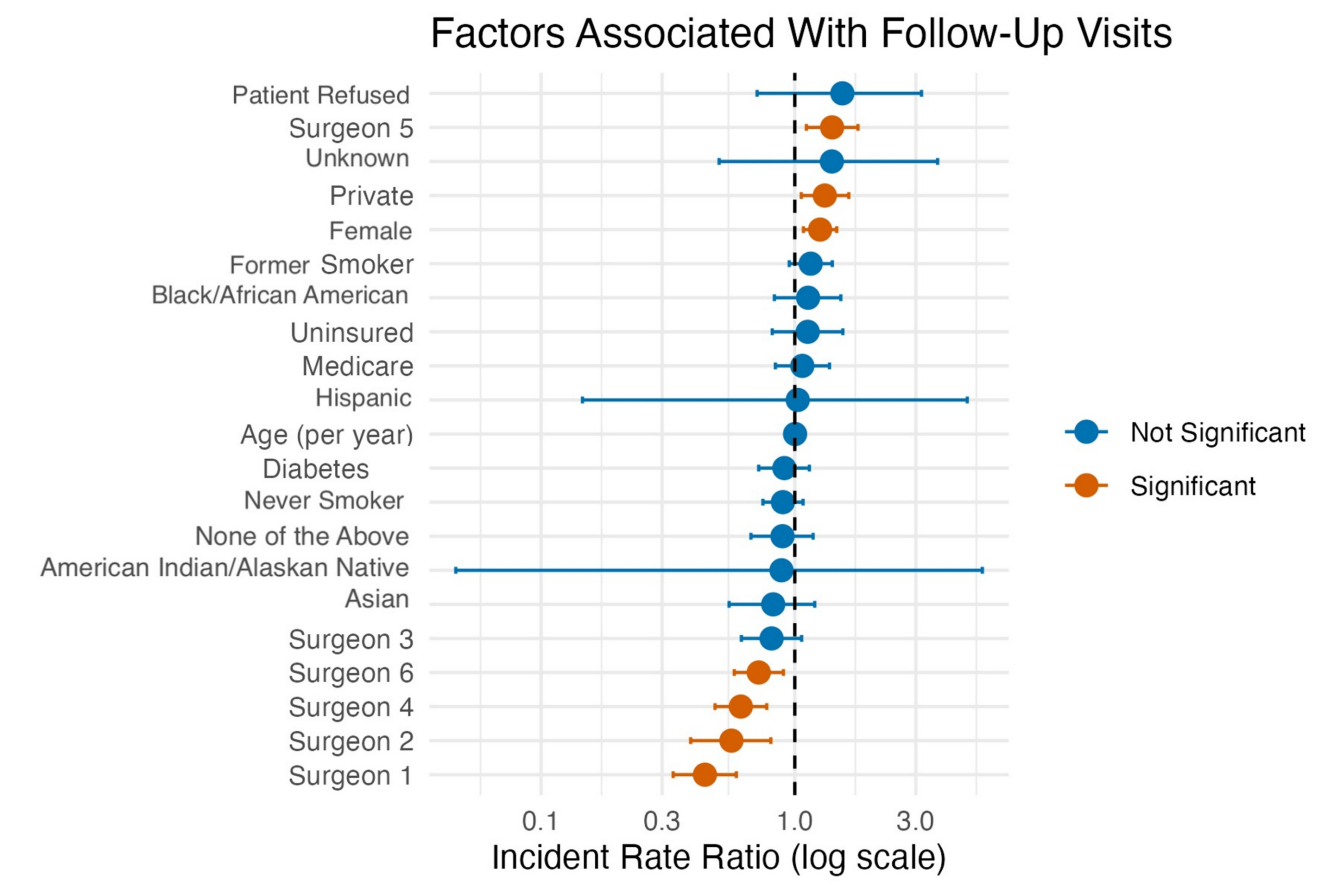
Forest plot of incidence rate ratios from multivariable negative binomial regression identifying predictors of postoperative follow-up utilization after distal radius open reduction and internal fixation

Multivariable negative binomial regression identified several independent predictors of surgeon-specific follow-up frequency. These findings, including the IRRs for each variable, are summarized in Table 2 and visualized in the forest plot (Figure 2). Surgeon identity was the strongest independent predictor of follow-up utilization, with marked variation observed across providers. These results represent statistical associations within one health system; due to the retrospective nature of the data and unmeasured differences in practice infrastructure (e.g., varying levels of APP integration), these findings do not imply causal relationships regarding the clinical necessity of any specific visit volume. Compared with the reference group ("Other Surgeons"), Surgeon 5 demonstrated significantly higher follow-up utilization, whereas Surgeons 1, 2, 4, and 6 demonstrated significantly fewer postoperative follow-up visits. Female gender was independently associated with a 26% increase in postoperative follow-up utilization with their surgeon (IRR: 1.26; 95% CI: 1.08-1.46; p=0.003). Private insurance was also associated with 31% higher follow-up rates with their surgeon compared with Medicaid (IRR: 1.31; 95% CI: 1.06-1.63; p=0.013). Age, race, diabetes status, smoking history, Medicare insurance, and uninsured status were not significantly associated with follow-up utilization with their operating surgeon after adjustment (all p>0.05). Model diagnostics suggested an adequate fit with minimal residual overdispersion (Pearson dispersion=1.13).

**Table 2:** Adjusted IRRs from multivariable negative binomial regression identifying predictors of postoperative follow-up visit frequency ^1^Statistically significant values are designated with "*". IRR: incidence rate ratio

Variable	IRR	95% CI lower	95% CI upper	z-statistic	P-value^1^
Female	1.26	1.08	1.46	2.997	0.003*
Black/African American	1.13	0.83	1.52	0.783	0.434
None of the above	0.89	0.67	1.18	-0.079	0.432
Hispanic	1.03	0.15	4.78	0.032	0.975
Asian	0.82	0.55	1.2	-1.001	0.317
American Indian/Alaskan Native	0.89	0.05	5.49	-0.110	0.912
Patient refused	1.54	0.71	3.16	1.142	0.253
Unknown	1.4	0.5	3.66	0.678	0.498
Age	1	1	1.01	0.937	0.349
Diabetes	0.91	0.72	1.14	-0.807	0.42
Never smoker	0.9	0.75	1.08	-1.166	0.244
Former smoker	1.16	0.95	1.41	1.449	0.147
Surgeon 1	0.44	0.33	0.59	-5.584	< 0.001*
Surgeon 2	0.56	0.39	0.8	-3.090	0.002*
Surgeon 3	0.81	0.62	1.06	-1.512	0.131
Surgeon 4	0.61	0.48	0.78	-4.090	< 0.001*
Surgeon 5	1.4	1.11	1.77	2.836	0.005*
Surgeon 6	0.72	0.58	0.9	-2.876	0.004*
Medicare	1.07	0.84	1.37	0.551	0.582
Private insurance	1.31	1.06	1.63	2.494	0.013*
Uninsured	1.12	0.81	1.55	0.723	0.47

Postoperative complications occurred in 53 patients (7.3%) (Table 1). The most common reported complications included nerve and tendon-related issues. The frequency of complications in the cohort was low. However, postoperative complications were strongly associated with follow-up frequency. Patients who experienced complications had 2.47 times as many postoperative follow-up visits compared with those without complications (IRR: 2.47; 95% CI: 2.14-2.85; p<0.001). But complication rates alone cannot fully account for the observed variability in follow-up utilization.

## Discussion

Summary of key findings

This study shows that postoperative follow-up utilization after distal radius ORIF varied widely across the cohort and was influenced by both patient- and provider-level factors. Surgeon practice patterns, serving as a proxy for both individual clinical habit and practice infrastructure, were the strongest independent predictor, with IRRs ranging from 0.44 to 1.40 compared with the reference group, corresponding to follow-up frequencies approximately 56% lower to 40% higher than the reference. Female gender (IRR: 1.26) and private insurance (IRR: 1.31) were also associated with higher utilization. Traditional clinical risk factors, including age, race, diabetes status, and smoking status, were not significantly associated with follow-up frequency. 

Patient-level predictors

Female patients had 26% higher follow-up visit counts than male patients, and privately insured patients had 31% higher follow-up visit counts than Medicaid patients. Female gender and private insurance were associated with higher postoperative follow-up utilization. Higher follow-up among women is consistent with prior reports in orthopedic and general surgery populations and may reflect greater health-seeking behavior or adherence to medical recommendations [9,20]. Similarly, privately insured patients had more follow-up visits, likely due to differences in access, scheduling flexibility, or financial barriers compared with publicly insured or uninsured patients [9,13,20-23]. Private insurance has been reported to have a protective effect in orthopedic trauma patients beyond the immediate postoperative period, while patients with additional socioeconomic factors cluster with insurance status and sex to compound disparities [9,12,24,25]. The lack of association with traditional clinical risk factors and follow-up frequency suggests that routine postoperative visit schedule intensity may not be fully explained by patient medical risk alone and may instead be tied to patient-level sociodemographic factors independently.

Surgeon-level predictors

Provider-level variability was the strongest predictor of follow-up utilization, even after adjustment for patient-level characteristics. Surgeon-specific IRRs ranged from 0.44 to 1.40 relative to the reference group, reflecting substantial variation in postoperative follow-up practices. Although limited literature directly evaluates surgeon-related determinants of follow-up after distal radius ORIF, prior work in hand and upper extremity surgery has demonstrated meaningful surgeon-driven variation in postoperative care protocols [17-19]. These findings suggest that provider practices, rather than patient clinical complexity alone, significantly influence postoperative utilization patterns.

However, these findings must be interpreted within the context of clinical practice infrastructure. Surgeon-level variability likely reflects differences in team-based care models rather than purely individual clinical decision-making. For instance, infrastructure was heterogeneous across our cohort: one surgeon lost APP support mid-study, while another practiced without APP support entirely. Additionally, some surgeons conducted informal postoperative check-ins during physical therapy visits, which are encounters that are not reliably captured in the electronic health record. Because our primary outcome was restricted to surgeon-only visits, surgeons who utilize high levels of APP-mediated care may appear to have "lower" utilization in this dataset, when in fact their patients may have received a higher total volume of clinical contact.

This heterogeneity of surgeon workflows serves as both a potential confounder and a central finding of our study. The observation that shifts in infrastructure (e.g., the loss of APP support) significantly alter follow-up counts suggests that utilization is often driven by "practice logistics" rather than evidence-based clinical necessity.

By approximately six weeks postoperatively, most patients demonstrated substantial recovery in basic functional tasks, with residual stiffness commonly managed through continued physical therapy. In this context, additional surgeon-directed visits may provide limited incremental value for uncomplicated cases, potentially explaining why some providers transition follow-up to APP-mediated care or therapy-based monitoring beyond this time point. Understanding how recovery trajectories intersect with provider workflow remains essential for developing evidence-informed, risk-stratified follow-up pathways that balance patient safety with efficient resource utilization.

Complications and clinical relevance

Complication rates in this cohort were low (7.3%) and did not fully explain differences in follow-up utilization. As expected, patients experiencing complications had higher follow-up counts, reflecting the need for closer monitoring. While complications in orthopedic hand surgery are well-documented, few studies have directly quantified how specific complication types translate into additional visits, imaging, or interventions, leaving a gap in the literature regarding their precise impact on follow-up utilization [5,17,26,27]. The overall variability between surgeons and across patient demographic factors remained evident even after accounting for complications, indicating that follow-up patterns are influenced by both clinical events and provider-level practice preferences. However, given the retrospective nature of this study, these findings support associations but do not allow for causal inferences regarding the impact of specific follow-up schedules on complication detection or mitigation.

Limitations and strengths

This study has several limitations. Its retrospective design precludes causal inference, and postoperative visits occurring outside our health system may not have been captured. Because the cohort was derived from a single regional tertiary care network, the findings may reflect local practice patterns and may not be fully generalizable to other healthcare settings. Functional outcomes and validated patient-reported outcome measures were not available within the EMR, limiting the assessment of the clinical impact of follow-up variability.

Crucially, the exclusion of APP-led visits and the lack of adjustment for practice infrastructure represent significant sources of potential bias. By defining follow-up strictly as surgeon-directed visits, we may have underestimated the total clinical surveillance provided to patients in team-based models. This narrow outcome definition likely inflates the observed surgeon-level variation, as a portion of this variability may simply reflect a transfer of care to APPs rather than a true difference in patient monitoring. Consequently, our findings should be viewed as an analysis of surgeon-level utilization rather than a comprehensive assessment of all postoperative points of contact.

Despite these limitations, the large sample size, use of negative binomial regression modeling appropriate for count data, and minimal residual overdispersion (Pearson dispersion=1.13) support the internal validity of our findings. These results are likely most applicable to tertiary care teaching hospitals and level I trauma centers with similar practice structures.

## Conclusions

Postoperative follow-up utilization after distal radius ORIF is significantly influenced by surgeon-level factors and practice infrastructure, as well as patient-level sociodemographic characteristics. While our study identifies wide variability in surgeon-directed visits, the retrospective design and absence of functional outcomes preclude a determination of which follow-up frequency is clinically superior. These findings suggest that current follow-up patterns are driven largely by practice logistics and provider habit rather than standardized, evidence-based protocols. Future prospective research incorporating patient-reported outcomes is necessary to establish optimal surveillance guidelines that balance clinical safety with efficient resource utilization.
